# Human-Manipulator Interface Using Particle Filter

**DOI:** 10.1155/2014/692165

**Published:** 2014-03-16

**Authors:** Guanglong Du, Ping Zhang, Xueqian Wang

**Affiliations:** South China University of Technology, Higher Education Mega Center, Guangzhou 510006, China

## Abstract

This paper utilizes a human-robot interface system which incorporates particle filter (PF) and adaptive multispace transformation (AMT) to track the pose of the human hand for controlling the robot manipulator. This system employs a 3D camera (Kinect) to determine the orientation and the translation of the human hand. We use Camshift algorithm to track the hand. PF is used to estimate the translation of the human hand. Although a PF is used for estimating the translation, the translation error increases in a short period of time when the sensors fail to detect the hand motion. Therefore, a methodology to correct the translation error is required. What is more, to be subject to the perceptive limitations and the motor limitations, human operator is hard to carry out the high precision operation. This paper proposes an adaptive multispace transformation (AMT) method to assist the operator to improve the accuracy and reliability in determining the pose of the robot. The human-robot interface system was experimentally tested in a lab environment, and the results indicate that such a system can successfully control a robot manipulator.

## 1. Introduction

Human intelligence is required to make a decision and control the robot especially when it is in unstructured dynamic environments. Thus, robot teleoperation is necessary in this situation especially when objects are unfamiliar and shapeless. There are some human-robot interfaces [1] like joysticks [2–4], dials, and robot replicas, and they have been commonly used. However, for completing a teleoperation task, these contacting mechanical devices always require unnatural hand and arm motion.

There is another way to communicate complex motions to a remote robot and it is more natural compared with using contacting mechanical devices. This method uses inertial sensors, contacting electromagnetic tracking sensors, gloves instruments with angle sensors, and exoskeleton systems [5] to track the operator hand-arm motion which completes the required task. However, these contacting devices may hinder natural human-limb motion.

Because vision-based techniques are noncontacting, they seldom hinder the hand-arm motion. Vision-based methods often use physical markers which are placed on the anatomical body part [6–8]. There are a lot of applications [6, 9, 10] using this marker-based tracking method. However, because body markers may hinder the motion for some highly dexterous tasks, operators may get occluded. Thus, this marker-based tracking method is not always practical. Due to this reason, a markerless approach seems better for many applications.

Compared to image-based tracking method which uses markers, markerless method not only is less invasive, but also eliminates problems about marker occlusion and identification [11]. Thus, for remote robot teleoperation, markerless tracking may be a better approach. However, existing markerless human-limb tracking techniques have a lot of limitations so that they may be difficult to use in robot teleoperation applications. Many existing markerless-tracking techniques capture images and then compute the motion later [12–15]. Thus, the robot manipulator can be controlled by continuous robot motion using the markerless tracking method. To allow the human operator to perform hand-arm motions for a task in a natural way without any interruption, the position and orientation of the hand and arm should be provided immediately. Many techniques can only provide 2D image information of the human motion [16, 17]; thus the tracking methods cannot be extended for accurate 3D joint-position data. An end-effector of a remote robot requires the 3D position and orientation information of the operator's limb-joint centers. How to identify human body parts in different orientations has always been a main challenge [12, 13, 18].

Some limited research towards markerless human-tracking has been done for robot teleoperation. Some use a human-robot interface based on hand-gesture recognition to control the robot motion [19–21]. Ionescu et al. [22] developed markerless hand-gesture recognition methods which can be used for mobile robot control and only need a few different commands such as “go,” “stop,” “left,” and “right.” However, for object manipulation in 3D space, it is not possible to achieve natural control and flexible robot motion by only gestures. If an operator wants to use gesture recognition method, he/she needs to think of those limited separate commands which human-robot interface can understand such as move up, down, and forward. A better way of human-robot interaction would be allowing the operator to focus on the complex global task and complete the task naturally when grasping and manipulating objects in 3D space instead of thinking about what type of hand motions is required.

To achieve this goal, a method that allows the operator to complete the task using natural hand-arm motions has to provide the robot manipulator with information of the hand-arm motion in real time. The information includes the hand and arm anatomical position and orientation [6, 23]. In [23], operators are required to use bare hands to control the robot and the accuracy is not enough to cope with the high-precision manipulation. Method [6] uses stereo vision to measure the human hand for controlling the robot. But the precision of the stereo vision is not so good and an occlusion is encountered easily. What is more, because method [6] needs the operator to make the big movements to carry out the task, it is time-consuming.

The proposed system uses a 3D Camera to locate the hand of the human operator and a camera to measure the distance between the robot tool and the target. This paper proposes Camshift to track the human hand and PF to estimate the position of the hand, as well as an adaptive multispace transformation method to improve the accuracy and efficiency of manipulation. Experimental results to validate the proposed methods are also presented.

The remainder of the paper is organized as follows. In Section 2, overview of our paper is presented. The human hand tracking system is then detailed in Section 3.  Section 4 describes the position estimation using PF. The method of adaptive multispace transformation is presented in Section 5. Experiments and results are presented in Section 6. Discussions are detailed in Section 7, followed by concluding remarks in Section 8.

## 2. Overview

**Figure 1:**
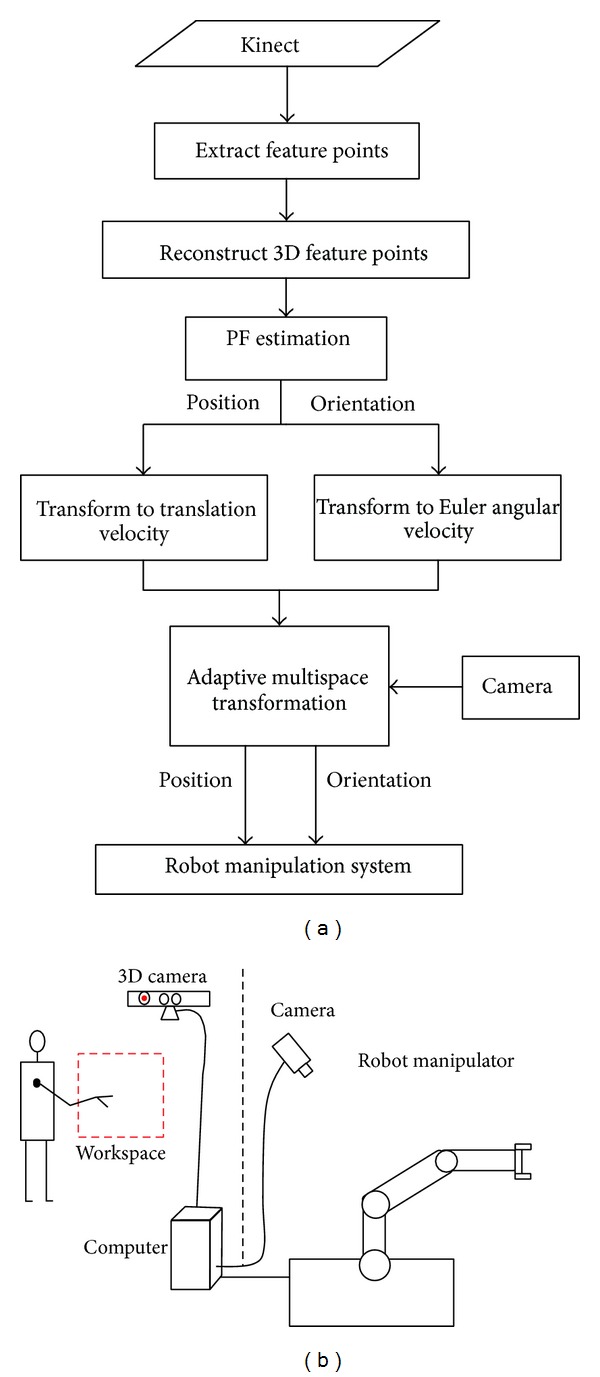
(a) Noninvasive robot teleoperation system. (b) Human-Robot interface system process.

Figure 1 shows the structure and data flow of this system. Fifteen Skeleton points are extracted from Kinect. A PF estimation algorithm is used to estimate the positions and the orientation of the hand. The positions and the orientations are processed via AMT to improve the accuracy of manipulation.

## 3. Human Hand Position Tracking

**Figure 2:**
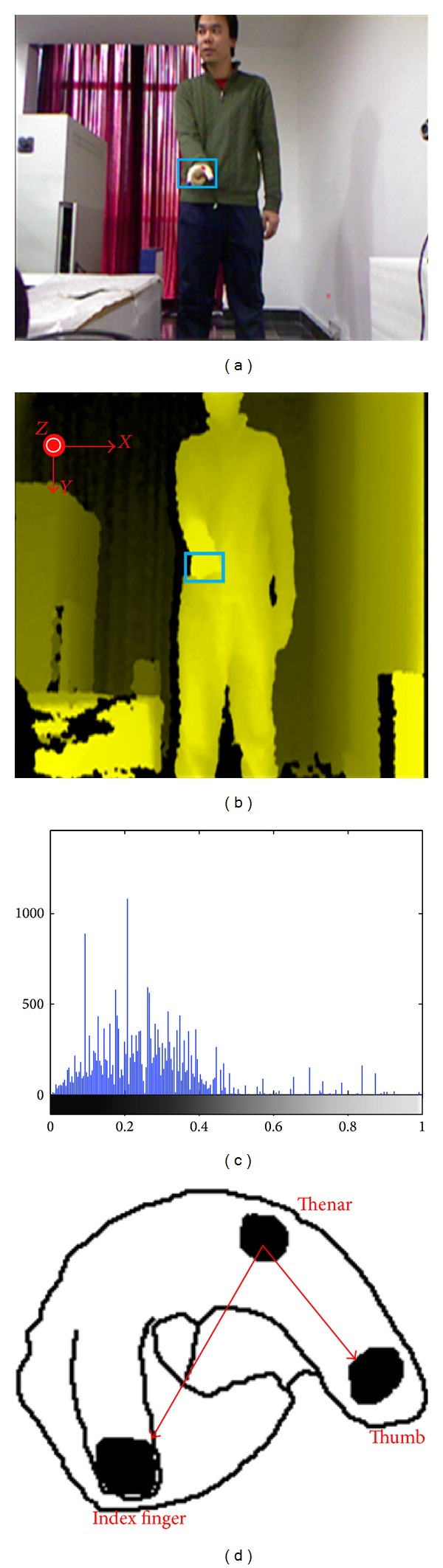
(a) Color image of the operator. (b) Depth image of the operator. (c) Histogram of selected color. (d) Feature points.

Since operators are required to wear a glove to control the robot, the position information of the marks on the gloves can be used to achieve the position information of human hands. Besides, the color of the marks makes the marks easier to identify. In the marks tracking system, we use 3D camera (Kinect) to obtain two 2D images of the IMU: color image (Figure 2(a)) and depth image (Figure 2(b)). And the marker positions are tracked by Camshift algorithm, which becomes more and more popular because of its performance in reality and robust [24].

Camshift algorithm employs a nonparameter method and use a clustering method to search targets. Camshift algorithm uses color information of the image and finds the target by color matching. Because the objects are very similar in the image sequences, the Camshift algorithm is very robust in this situation. Camshift tracking system includes color probability distribution and Camshift flow.

### 3.1. Color Probability Distribution

Because of the fact that hue, saturation, and brightness are separate in HSV color space, the stability of the algorithm will increase by using HSV space. In this paper, the value of hue is selected as the parameter to build target histogram. The transformation equations from RGB space to HSV space are

(1) H={π3(g−bmax⁡(r,g,b)−min⁡(r,g,b)) if  (r=max⁡(r,g,b)),π3(2+b−rmax⁡(r,g,b)−min⁡(r,g,b)) if  (g=max⁡(r,g,b)),π3(4+r−gmax⁡(r,g,b)−min⁡(r,g,b)) if  (b=max⁡(r,g,b)),S=max⁡(r,g,b)−min⁡(r,g,b)max⁡(r,g,b),V=max⁡(r,g,b).

Let the total number of image pixel be *n*, let the level of histogram of hue be *m*, let the image pixel be *x*_*i*_, and let the histogram index function of *x*_*i*_ be *c*(*x*_*i*_); then the object histogram of hue can be described as

(2) $$\begin{array}{c} q_{u}=\sum_{i=1}^{n}\delta \left(c\left(x_{i}\right)-u\right)\;u=1,\ldots ,m, \end{array}$$

where *δ* is unit impulse transfer function.  Figure 2 shows the color image of the selected region and the corresponding hue histogram.

### 3.2. Camshift Flow

After establishing the color probability model of the object, every video frame should be converted into a color probability distribution image *I*. The process of Camshift flow is shown as follows.

(1) Select search window with the size of *s* in *I* and determine the original centre position of the search window.
(2) Calculate the 0th and 1st order moment:
  (3) $$\begin{array}{c} M_{00}=\sum_{x}\;\sum_{y}I\left(x,y\right),\\ M_{10}=\sum_{x}\;\sum_{y}xI\left(x,y\right),\\ M_{01}=\sum_{x}\;\sum_{y}yI\left(x,y\right). \end{array}$$
(3) Calculate the mass centre of the search window
  (4) $$\begin{array}{c} x_{c}=\frac{M_{10}}{M_{00}},\\ y_{c}=\frac{M_{01}}{M_{00}}. \end{array}$$
(4) Set the centre of the window as the mass centre and repeat steps 2, 3 until the mass centre position converges to a point.
(5) Calculate the 2th order moments:
  (5) $$\begin{array}{c} M_{20}=\sum_{x}\;\sum_{y}x^{2}I\left(x,y\right),\\ M_{02}=\sum_{x}\;\sum_{y}y^{2}I\left(x,y\right),\\ M_{11}=\sum_{x}\;\sum_{y}xyI\left(x,y\right). \end{array}$$
(6) Calculate parameters (*a*, *b*, *c*):
  (6) $$\begin{array}{c} a=\frac{M_{20}}{M_{00}}-x_{c}^{2},\\ b=2\left(\frac{M_{11}}{M_{00}}-x_{c}y_{c}\right),\\ c=\frac{M_{02}}{M_{00}}-y_{c}^{2}. \end{array}$$
(7) Calculate the size of major and minor axis of tracked object and direction angle of ellipse in image:
  (7) $$\begin{array}{c} \theta =\frac{1}{2}\text{ta}{\text{n}}^{-1}\left(\frac{b}{a-c}\right),\\ w=\sqrt{\frac{\left(a+c\right)-\sqrt{b^{2}+{\left(a-c\right)}^{2}}}{2}},\\ l=\sqrt{\frac{\left(a+c\right)+\sqrt{b^{2}+{\left(a-c\right)}^{2}}}{2}}. \end{array}$$

Then the new window from the calculated ellipse can be achieved and the window should be set as the original window in the next video frame.

### 3.3. Feature Points Extracting

The feature points from the window are determined by Camshift. In the feature points extracting system, RGB color model is required. In 24-bit RGB color model, every color is represented by red, green, and blue. Different combination yields different color. The glove used in this paper is yellow, and the marker is red and blue. The tracking program needs to recognize the red and blue point in the image to extract the human hand.

Assume the value of a pixel in the image is (*R*_*i*_, *G*_*i*_, *B*_*i*_); the model to recognize the red point in the image is as follow:

(8) Ri>Gi+δg,Ri>Bi+δb,Ri>δ,

where *δ*_*g*_, *δ*_*b*_, *δ* are the threshold values.

The 3D coordinates of the feature points are reconstructed by the depth images (Figure 2(b)).

## 4. Position Estimation for Feature Points Using PF

### 4.1. Particle Filter

The PF is a suboptimal resolution, which estimates the true posterior using a finite number of random state samples amongst their corresponding normalized weights. Thus, at time *t*_*k*_, the posterior density approximation is

(9) $$\begin{array}{c} p\left(x_{k}\;|\;z_{1:k},u_{0:k-1}\right)\approx \sum_{i=1}^{N}\omega_{k}^{i}\delta \left(x_{k}-x_{k}^{i}\right), \end{array}$$

where *δ*(·) is the Dirac delta function, *N* is the number of samples, *ω*_*k*_^*i*^ is the normalized weight of the *i*th particle, and *x*_*k*_^*i*^ is the *i*th particle.

For derivation of the PF state estimation algorithm, the posterior density up to time *t*_*k*_ is

(10) p(x0:k ∣ z1:k,u0:k−1)  =p(zk ∣ xk)·p(xk ∣ xk−1,uk−1)   ·p(x0:k−1 ∣ z1:k−1,u0:k−2)   ×(p(zk ∣ z1:k,u0:k−1))−1.

Since sampling from the posterior density is frequently difficult, an importance sampling technique [25] is employed. If target density (posterior density in this case) can be assessed at any juncture, but sampling remains difficult, samples may be obtained from a recognized normalized probability density [*r*(*x*)], the so-called importance density. In order to counteract for variances between the target and the importance densities, normalized weights defined as the ratios of the two densities are given to each particle [26]. Up to time *t*_*k*_, the discrete posterior density approximation is described as

(11) $$\begin{array}{c} p\left(x_{0:k}\;|\;z_{1:k},u_{0:k-1}\right)\approx \sum_{i=1}^{N}\omega_{k}^{i}\delta \left(x_{0:k}-x_{0:k}^{i}\right) \end{array}$$

The normalized weight, defined in (12), possesses the subsequent correlation with the target and importance densities, *p*(*x*) and *r*(*x*), respectively:

(12) ωki∝p(x0:ki ∣ z1:k,u0:k−1)r(x0:ki ∣ z1:k,u0:k−1)∝p(zk ∣ xki)·p(xki ∣ xk−1i,uk−1)·p(x0:k−1i ∣ z1:k−1,u0:k−2)×(p(x0:ki ∣ z1:k,u0:k−1))−1.

Note that the importance density must be selected such that it may be determined recursively as

(13) r(x0:k ∣ z1:k,u0:k−1)=r(xk ∣ x0:k−1,z1:k,u0:k−1)·r(x0:k−1 ∣ z1:k−1,u0:k−2).

If the importance density, like the target density, also fulfills the Markov property, (12) can be rewritten as

(14) ωki∝p(zk ∣ xki)·p(xki ∣ xk−1i,uk−1)·p(x0:k−1i ∣ z1:k−1,u0:k−2)×(r(xki ∣ xk−1i,zk,uk−1)  ·r(x0:k−1i ∣ z1:k−1,u0:k−2))−1∝p(zk ∣ xki)·p(xki ∣ xk−1i,uk−1)r(xki ∣ xk−1i,zk,uk−1)·ωk−1i.

With further simplification of (14), the importance density can be selected from prior as

(15) $$\begin{array}{c} r\left(x_{k}\;|\;x_{k-1},z_{k},u_{k-1}\right)=p\left(x_{k}\;|\;x_{k-1},u_{k-1}\right). \end{array}$$

Subsequently, (14) can be rewritten as

(16) $$\begin{array}{c} \omega_{k}^{i}\propto \omega_{k-1}^{i}\cdot p\left(z_{k}\;|\;x_{k}^{i}\right). \end{array}$$

This type of PF is problematic in that, when *k* is high, only one particle will possess higher weights (nearing unity), while the weights of all remaining particles will be negligible (approaching zero). This phenomenon, referred to as the degeneracy problem, is unfavorable since the weighted particles fail to accurately reflect the real posterior density. To circumvent this issue, resampling of particles on the basis of their weights should be performed. After resampling, all particles are assigned the same weight; the weights at time *t*_*k*−1_ are identical (*ω*_*k*_^*i*^ = 1/*N*). Resampling reduces the sample numbers from the lower weights by drawing more higher-weight samples. Consequently, (16) becomes

(17) $$\begin{array}{c} \omega_{k}^{i}\propto p\left(z_{k}\;|\;x_{k}^{i}\right). \end{array}$$

Furthermore, after resampling at time *t*_*k*_, (11) can be written as

(18) $$\begin{array}{c} p\left(x_{k}\;|\;z_{1:k},u_{0:k-1}\right)\approx \frac{1}{N}\sum_{i=1}^{N}\delta \left(x_{k}-x_{k}^{i}\right). \end{array}$$

Various methods reported in the literature propose calculating weights on the basis of “fitness” [27, 28] or “evidence” [29, 30] values of each particle to characterize the likelihood.

### 4.2. Position Estimation Using PF

This proposed method estimates the object's position using a PF, whose state is set as [*x*_PF_ = (*p*_*x*_, *p*_*y*_, *p*_*z*_)]. The position measurements can be used as the expected value and the position calculation of each position particle can be used to determine the weight of the particle. However, in an approach like this, differences for each position state in the position measurements and position calculation are not only due to the position state, but also due to noise from sensor errors.

For a time period (Δ*Ts*, where subscript *s* is the *s*th orientation iteration, *s* = 1,2,…), the summation of the position difference is used to obtain the weights rather than the immediate position difference at time *t*_*k*_. Then, the accumulated position difference from the estimated and calculated values for the *i*th particle is used in the likelihood calculation as in

(19) $$\begin{array}{c} PE_{s}^{i}=\sum_{k=\left(s-1\right)\cdot M_{S}+1}^{M_{S}\cdot s}\;\sum_{j=(x,y,z)}{\left[P_{p-j,k}^{i}-P_{j,k}^{i}\right]}^{2}, \end{array}$$

where *Ms* = Δ*Ts*/*t*, *P*_axis,*k*_^*i*^ is the measured position component and *P*_*p*-axis,*k*_^*i*^ are the position states. For a given particle, the lower the *E*_*s*_^*i*^, the greater the likelihood that the orientation is correct. Based on the *PE*_*s*_^*i*^ values the weight of each particle is recalculated for every Δ*Ts* period that elapses. Then, rather than using the direct position measurements, the PF should be altered to use *PE*_*s*_^*i*^. So, up to time *t*_*s*_ the posterior approximation becomes

(20) $$\begin{array}{c} p\left(x_{0:s}\;|\;PE_{1:s}^{i}\right)\approx \sum_{i=1}^{N}\omega_{k}^{i}\delta \left(x_{0:s}-x_{0:s}^{i}\right). \end{array}$$

Then, for up to time *t*_*s*_ the posterior can be presented as

(21) p(x0:s ∣ PE1:si)∝p(PEsi ∣ xs)·p(xs ∣ xs−1)·p(x0:s−1 ∣ PE1:s−1i).

And at the *s*th orientation iteration the normalized weight is

(22) $$\begin{array}{c} \omega_{s}^{i}\propto \frac{p\left(PE_{s}^{i}\;|\;x_{s}^{i}\right)\cdot p\left(x_{s}^{i}\;|\;x_{s-1}^{i}\right)}{r\left(x_{s}^{i}\;|\;x_{s-1}^{i},PE_{s}^{i}\right)}\cdot \omega_{s-1}^{i}. \end{array}$$

The following relationship for the normalized weight is determined through resampling and picking the importance density from *p*(*x*_*s*_ | *x*_*s*−1_):

(23) $$\begin{array}{c} \omega_{s}^{i}\propto p\left(PE_{s}^{i}\;|\;x_{\text{PF},s}^{i}\right). \end{array}$$

Basing the calculation of *PE*_*s*_^*i*^, the particle's weight is determined as the most likely value of *PE*_*s*_^*i*^. argmin(*PE*_*s*_^*i*^) is used as the orientation with the smallest accumulated position error that is most likely to have the correct orientation. This means that the calculation for the normalized weight is

(24) $$\begin{array}{c} \omega_{s}^{i}\propto \exp\left(\frac{-{\left(PE_{s}^{i}-\text{argmin}\left(PE_{s}^{i}\right)\right)}^{2}}{2\times {\left(\sigma \left(PE_{s}^{i}\right)\right)}^{2}}\right), \end{array}$$

where the standard deviation of *PE*_*s*_^*i*^ is *σ*(*PE*_*s*_^*i*^). The proposed particle filtering technique can be summarized and described as follows.

*Step  1.* Initialization: if *k* = 1, draw *x*_0_^*f*^ from *p*(*x*_0_) and *ω*_0_^*i*^ = 1/*N*.

*Step  2.* Prediction: calculate *x*_PF,*k*_^*i*^ according to (18).

*Step  3.* Likelihood: calculate *PE*_*s*_^*i*^ according to (19).

*Step  4.* Update: if it remains that (*k*/*M*_*s*_) = 0, then

(1) predicted states *x*_*s*_^*i*^ = *x*_PF,*k*_^*i*^;
(2) calculate
  (25) $$\begin{array}{c} \omega_{s}^{*i}=\exp\left(\frac{-{\left(PE_{s}^{i}-\text{argmin}\left(PE_{s}^{i}\right)\right)}^{2}}{2\times {\left(\sigma \left(PE_{s}^{i}\right)\right)}^{2}}\right); \end{array}$$
(3) weight calculation:
  (a) normalize weights *ω*_*s*_^*i*^ = *ω*_*s*_^∗*i*^/∑_*i*=1_^*N*^ *ω*_*s*_^∗*i*^
  (b) resampling Draw *x*_*s*_^*i*^ based on *ω*_*s*_^*i*^
  (c) reset weights *ω*_*s*_^*i*^ = 1/*N*
  (d) *x*_PF,*k*_^*i*^ = *x*_*s*_^*i*^

Over.

### 4.3. Pose of Hand

**Figure 3:**
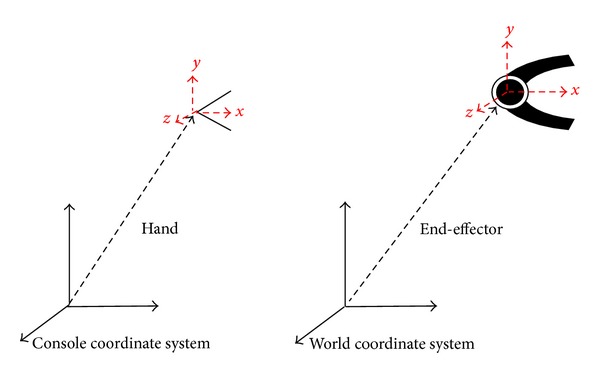
Orientation Model.

In this paper, the position of the marked points in the glove is used to estimate the position of the human hand. The orientation of the hand is in accordance with the orientation formed by thumb tip, index finger tip, and palm space of the operator's hand (Figure 3).

It means once the transformation matrix is obtained, which is a transformation matrix from the coordinate system of the console to the coordinate system of the operator's hand. The transformation matrix from the base coordinate system to the end-effector is also obtained. The derivation of the orientation matrix is detailed below.

Assume the origin of the operator's hand coordinate system is identical to the one in the console coordinate system, and the transformation matrix is a 3∗3 matrix *M*. In the process of hand tracking and positioning, the unit vector [*x*_1_, *x*_2_, *x*_3_], [*y*_1_, *y*_2_, *y*_3_], [*z*_1_, *z*_2_, *z*_3_] in direction *X*, *Y*, *Z* which are measured by cameras yield is

(26) $$\begin{array}{c} \left[\begin{array}{ccc} m_{11} & m_{12} & m_{13}\\ m_{21} & m_{22} & m_{23}\\ m_{31} & m_{32} & m_{33} \end{array}\right]\left[\begin{array}{c} 1\\ 0\\ 0 \end{array}\right]=\left[\begin{array}{c} x_{1}\\ x_{2}\\ x_{3} \end{array}\right],\\ \left[\begin{array}{ccc} m_{11} & m_{12} & m_{13}\\ m_{21} & m_{22} & m_{23}\\ m_{31} & m_{32} & m_{33} \end{array}\right]\left[\begin{array}{c} 0\\ 1\\ 0 \end{array}\right]=\left[\begin{array}{c} y_{1}\\ y_{2}\\ y_{3} \end{array}\right],\\ \left[\begin{array}{ccc} m_{11} & m_{12} & m_{13}\\ m_{21} & m_{22} & m_{23}\\ m_{31} & m_{32} & m_{33} \end{array}\right]\left[\begin{array}{c} 0\\ 0\\ 1 \end{array}\right]=\left[\begin{array}{c} z_{1}\\ z_{2}\\ z_{3} \end{array}\right]. \end{array}$$

Through (17), it yields

(27) $$\begin{array}{c} \left[\begin{array}{ccc} m_{11} & m_{12} & m_{13}\\ m_{21} & m_{22} & m_{23}\\ m_{31} & m_{32} & m_{33} \end{array}\right]=\left[\begin{array}{ccc} \begin{array}{c} x_{1}\\ x_{2}\\ x_{3} \end{array} & \begin{array}{c} y_{1}\\ y_{2}\\ y_{3} \end{array} & \begin{array}{c} z_{1}\\ z_{2}\\ z_{3} \end{array} \end{array}\right]. \end{array}$$

As stated before, the transformation matrix from console coordinate system to operator's hand coordinate system is

(28) $$\begin{array}{c} M=\left[\begin{array}{cccc} x_{1} & y_{1} & z_{1} & p_{1}\\ x_{2} & y_{2} & z_{2} & p_{2}\\ x_{3} & y_{3} & z_{3} & p_{3}\\ 0 & 0 & 0 & 1 \end{array}\right]. \end{array}$$

Notice that the [*p*_1_, *p*_2_, *p*_3_] is the translation matrix of the hand.

## 5. Robot Manipulation System

### 5.1. Adaptive Multispace Transformation (AMT)

**Figure 4:**
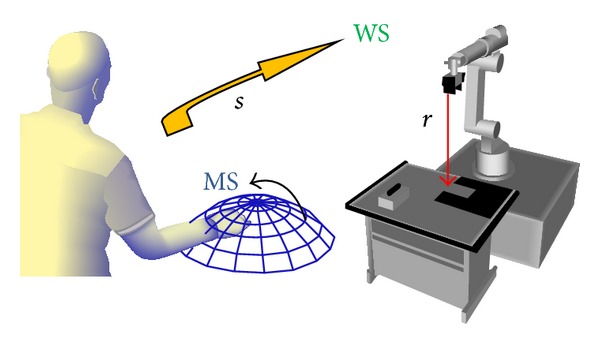
The structure of AMT.

Humans have inherent perceptive limitations (such as the perception of distance) and motor limitations (such as physiological tremor), which prevent them from operating precisely and smoothly enough for certain tasks [31]. To improve visual and practical performances of the teleoperation interface, a modified adaptive multispace transformation (AMT) is applied. This method involves an interface with two scaling processes which links human operators' workspace and robots' working space (Figure 4). One process scales the movement produced by the human operator. The other process changes the scale of the vector *K*, which is the virtual unit vector of the central axis of the robot EE and the robot movements. These changes help the operator to modify the robot speed and improve performance.

Scaling vector *S* is used to map the actions of the human hand from master space MS to the working space WS. *S* is a function of the distance *r* between robot EE and the target. When *S* < 1, it decelerates the movement of robot EE. Otherwise, it accelerates the movement of *K* when >1.

As the movement of the virtual position in VS is affected by vector *S*, the Euler angular velocity in MS ${\dot{O}}_{M}$ and the angular velocity in WS ${\dot{O}}_{W}$ are

(29) $$\begin{array}{c} {\dot{O}}_{W}=\{\begin{array}{cc} S\cdot {\dot{O}}_{M}, & {\dot{O}}_{M}<\tau_{o},\\ 0, & {\dot{O}}_{M}\geq \tau_{o}. \end{array} \end{array}$$

Assume that ${\dot{P}}_{M}$ is the velocity vector of the hand movement in MS and ${\dot{P}}_{W}$ is the speed of the robot EE in WS. So we have

(30) $$\begin{array}{c} {\dot{P}}_{W}=\{\begin{array}{cc} S\cdot {\dot{P}}_{M}, & {\dot{P}}_{M}\leq \tau_{p},\\ 0, & {\dot{P}}_{M}>\tau_{p}, \end{array} \end{array}$$

where *τ*_*o*_ and *τ*_*p*_ are the threshold. Since *S* is a function of distance *r*, the function *u*(*r*) is defined as

(31) $$\begin{array}{c} S\left(r\right)=\log\left(C_{1}*r\right)+C_{2}, \end{array}$$

where *C*_1_ and *C*_2_ are constants.

### 5.2. Robot Control

Moving angles of Robot's joints can be achieved by the solution of inverse problems. Given a position and pose of end-effector, every angle that makes a robot reach to the expected position can be achieved by the solution of the inverse problem.

In the standard DH representation, *A*_*i*_ presents the homogeneous coordinate transformation matrix from coordinate *i* − 1 to *i*:

(32) $$\begin{array}{c} A_{i}=\left[\begin{array}{cccc} \begin{array}{c} \cos\theta_{i}\\ \sin\theta_{i}\\ 0\\ 0 \end{array} & \begin{array}{c} -\sin\theta_{i}\cos\alpha_{i}\\ \cos\theta_{i}\cos\alpha_{i}\\ \sin\alpha_{i}\\ 0 \end{array} & \begin{array}{c} \sin\theta_{i}\sin\alpha_{i}\\ -\cos\theta_{i}\sin\alpha_{i}\\ \cos\alpha_{i}\\ 0 \end{array} & \begin{array}{c} l_{i}\cos\theta_{i}\\ l_{i}\sin\theta_{i}\\ r_{i}\\ 1 \end{array} \end{array}\right]. \end{array}$$

For a robot with 6 joints, the homogeneous coordinate transformation matrix from base coordinate system to end-effector's coordinate system is defined as

(33) $$\begin{array}{c} T_{6}=A_{1}A_{2},\ldots ,A_{6}=\left[\begin{array}{cccc} n_{6}^{0} & s_{6}^{0} & a_{6}^{0} & p_{6}^{0}\\ 0 & 0 & 0 & 1 \end{array}\right], \end{array}$$

where *n*_6_^0^ is the row vector of the end-effector, *s*_6_^0^ is the pitch vector, *a*_6_^0^ is the yaw vector, and *p*_6_^0^ is the position vector.

Using (32) and (28), it is known that

(34) $$\begin{array}{c} T_{6}=M. \end{array}$$

To solve the nonlinear (8), Levenberg-Marquardt (LM algorithm) [32] is used to fit (*θ*_1_, *θ*_2_,…, *θ*_6_) to the nonlinear model.

## 6. Experiments

### 6.1. Environment of Experiment

To verify the proposed method, a series of five tests are carried out to imitate the human hand motion in the human-robot-manipulator interfaces for robot teleoperation. A GOOGOL GRB3016 robot is used in our experiments.  Table 1 lists the nominal Denavit-Hartenberg (DH) [33] parameters of the robot. We compared our method with the method [6] in the accuracy of these two methods which has been tested as well as the ability of the manipulator to imitate human hand-arm motion. Being different from method [6], which controls the position and orientation of robot manipulators directly, our method uses velocity control. In every test, the operator moved his arm in the working space to perform a pick-and-place task which requires the operator to pick up an object and place it on a target position whose edges were aligned as shown in Figure 6.

**Figure 5:**
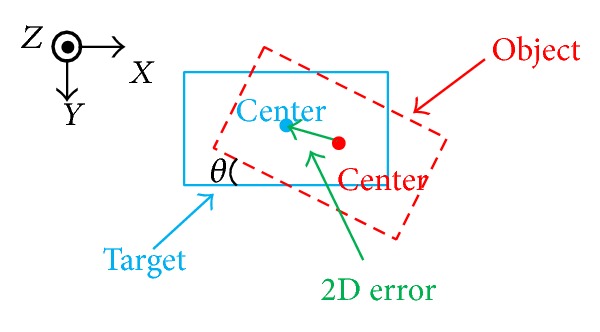
Definition of errors.

**Figure 6:**
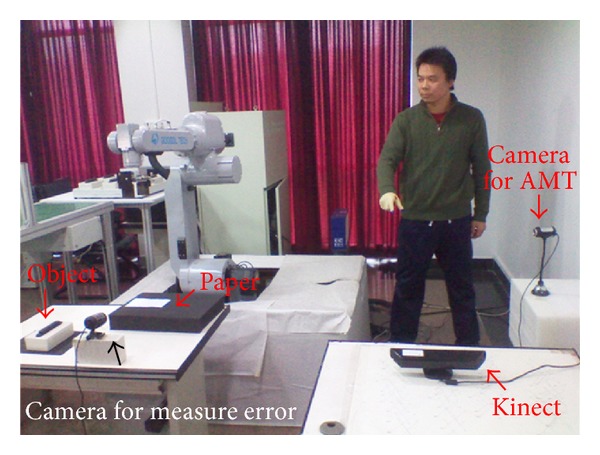
Environments of experiment.

Because picking up and placing the object require opening or closing the claws of the robot, we use a motion to give this order. When the distance of two blue points is small enough, the claws will be close. Instead, when the distance of two blue points is large enough, the claws will be open. The object used in the tests is a rigid plastic block whose height, width, and thickness are 180 mm, 90 mm, and 50 mm correspondingly. The 2D error *E*_pos,2D_ in translation is the distance between two center points of the target and object. The 2D error *E*_ori,2D_ in rotation is the angle *θ* between two edges of the target and the object (Figure 5).

### 6.2. Results of Experiment

**Figure 7:**
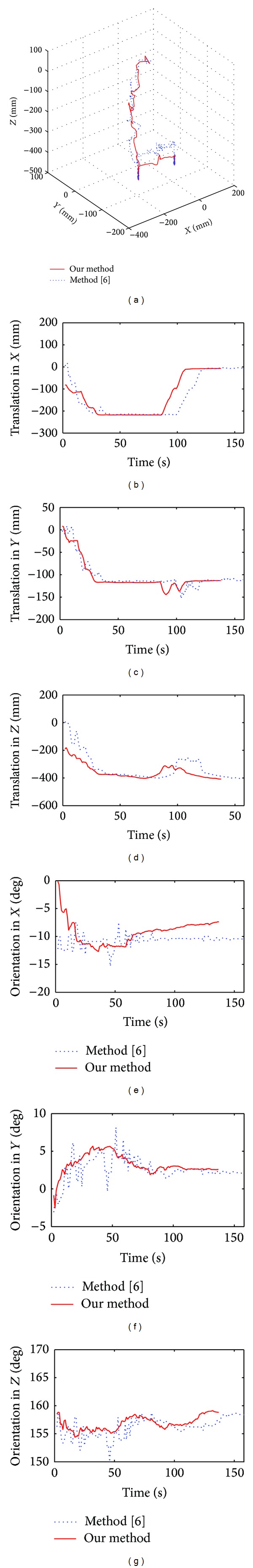
Results of test 3.

Figure 7 shows the results of the human hand and robot EE (Test 3). The 3D paths of the robot EE for two methods were shown in Figure 7(a). The path of our method is the red line and that of method [6] is the blue dotted line. Figures 7(b)–7(g) show the result of the translation and the orientation of the robot with method [6]. There are three circles on the 3D path of the robot EE (Figure 7(a)). The time from the beginning to the first circle is the period of approaching the object. The time from the first circle to the second circle is the period of picking the object. Then the time from the second circle to the third circle is the period of placing the object on the target. During the period of picking the object and the period of placing the object on the target by the robot, some minor correction of the position and orientation of the robot EE for overshoot was required, mainly preceding gripper closing to pick the object and gripper opening to place object on the target. Because of the hands shaking or body shaking, the curves at the stationary period are barely straight. During the object manipulation tasks, the operator needed to make frequent movement to adjust the orientations. With our method, the period of picking the object is 45th s to 84th s and 112th s to 136th for the period of placing. With method [6], the period of picking the object is 41th s to 97th s and 120th s to 157th s for the period of placing. The results with our method are rather excellent.

**Figure 8:**
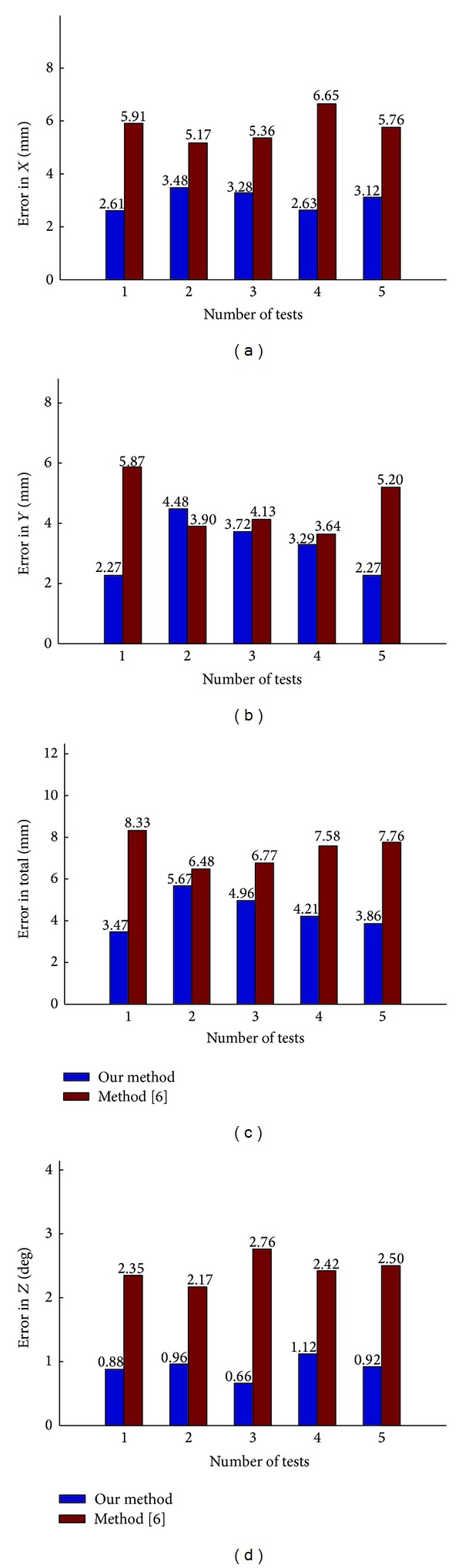
Robot EE errors for five tests.

In experiment, we compared our method with method [6] in terms of accuracy and operating time.  Figure 8 shows the errors for 5 tests with two methods. In our method, the 2D absolute errors for 5 tests ranged from 2.61 mm to 3.48 mm in *X* translation, 2.27 mm to 4.47 mm in *Y* translation, and 0.66 deg to 1.11 deg in *Z* rotation, with the mean absolute errors (MEs) of 3.02 mm, 3.20 mm, and 0.91 deg. In method [6], the 2D absolute errors for 5 tests ranged from 5.17 mm to 6.64 mm in *X* translation, 3.64 mm to 5.87 mm in *Y* translation, and 2.17 deg to 2.75 deg in *Z* rotation, with the mean errors (MEs) of 5.77 mm, 4.54 mm, and 2.44 deg. Comparing with method [6], the mean total errors of translation of our method drop by 2.94 mm, as well as the *Z* rotation errors drop by 1.53 deg. The translation errors of our method are slightly smaller than those of method [6], but the rotation errors of our method are less than half of those of method [6]. In our method, we used AMT to measure the orientation of the human hand, so the rotation errors are smaller in our method. But due to the perceptive limitations and the motor limitations, the human operator is hard to carry out the high precision operation.

Since the method [6] needs to execute big movements to control the robot manipulator, the operating time of method [6] is longer than that of our method (Table 2). The mean time of method [6] is 165.66 s, which is more than that of our method (131.0 s). With AMT method, the robot can move by a large range even though the operator makes small movements. Due to the accurate manipulation, the operator did not need to make so much correction of position and orientation and it saved a lot of time of operation.

## 7. Discussions

The robot is controlled by extracting the position and pose of human hands, including tips of index fingers, palms, and thumbs. In reality, the coordinate of human hands extracted by Camshift algorithm may be unstable and human hands would tremble sometimes due to physical limitation. Thus tremble phenomenon may occur in control data. This paper uses velocity control theory to eliminate the data jitter.

For the robot teleoperation in the remote unstructured environment, a condition is assumed that all the remote robot site components can be installed on a mobile platform and enter those unstructured environment. And the remote unstructured environments involve robotic arm, robot controller, cameras on end-effectors, and some other cameras. The method shown here is testified on grabbing, picking, and inserting a peg into a hole. This system includes the operator into the decision control loop and that is a significant advantage. Firstly, it allows a robot to finish some tasks like grabbing or picking objects without any prior knowledge like start location and even destination location. Secondly, there are some similar tasks requiring decision making. When picking objects or cleaning some objects which may contain some dangerous items, decision making is always required. Also it is expected that this system can be used to achieve those complex poses when the joints of the robot are limited. The peg-into-hole tasks testify the ability of this robot teleoperation method to determine the random position of the target and the object. This function is helpful because assembly and disassembly tasks include more limited peg-into-hole tasks. However, for these tasks, an appropriate grabhook, bigger hole, and groove are needed unless this system includes force feedback.

Compared with contacting electromagnetic devices, like hand joystick and data gloves, our method would not hinder most natural human-limb motion and allows the operator to concentrate on his own task instead of decomposing commands into some simple commands. Compared with the noncontacting markerless method [23], our method proves more accurate and stable. This system can be used immediately without any initialization. Compared with the automatic capture [6], this algorithm uses manual positioning. Considering the hand tremor, this algorithm includes coarse adjustment and fine adjustment function. When guiding the robot, we use the coarse adjustment to make the robot move close to the target fast. When grabbing the target, we use the fine adjustment to position the robot accurately. This can ensure the safety and the efficiency of the teleoperation and solve the problem of inaccuracy caused by manual operation.

This paper contributes to the guiding teleoperation system based on noncontacting measurement. By using tracking based on Kinect, now robot teleoperation allows the operator to control the robot by a more natural way. Generally speaking, using the same hand motion that naturally would be used in a task can accomplish the operation task. And what is more, this tracking based on Kinect is noncontact. Thus, compared with contacting electromagnetic devices, devices based on sensor and data gloves which are used normally, noncontacting devices may less hinder natural human-limb motion. The method raised here will allow the operator to focus on his task instead of thinking of how to decompose the commands into some simple commands that the voice recognition teleoperation system can understand. This method raised here is more natural and intuitive than the operation in [23]. The system can be used immediately without any initialization. And this noncontacting control system can be used outdoors.

Further research can merge the gesture control and voice control together. Simple commands like start, end, and pause can be controlled by voice. And there is no need to recognize the specific motion and thus this system can control the robot more flexibly and advantageously.

## 8. Conclusions

This paper presents a human-robot interface system that utilizes one 3D camera configuration PF and that is developed to estimate the position and the orientation of the human hand more stably. To eliminate the effect of the occlusion and the failure of motion sensing, an AMT method is proposed in this paper. Since humans have inherent perceptive limitations and motor limitations, the high-precision tasks cannot be performed directly by a human. Therefore, we employ an AMT system to assist the operator to perform the high-precision tasks.

In the experiment, a task of picking and placing and a task of peg-into-hold were carried out. We compared the accuracy and the efficiency between our method and method [6]. The experimental results show that our method has better performance.

## Figures and Tables

**Table 1:** The nominal link parameters in DH model for the GOOGOL GRB3016 robot.

Joint	DH			
	*a* (mm)	*α* (rad)	*d* (mm)	*θ* (rad)
1	150	−*π*/2	250	0
2	570	−*π*	0	−*π*/2
3	150	*π*/2	0	0
4	0	−*π*/2	650	0
5	0	−*π*/2	0	−*π*/2
6	0	0	−200	0

*a*: length of the common normal.

*α*: angle about common normal, from old axis to new axis.

*d*: offset along previous to the common normal.

*θ*: angle about previous, from old to new.

**Table 2:** Result of operation time.

Tests	1	2	3	4	5
Our method	136 s	128 s	136 s	118 s	137 s
Method [6]	166 s	155 s	163 s	171 s	173 s
